# Could paper package leaflet be left out from hospital products?

**DOI:** 10.1016/j.rcsop.2022.100176

**Published:** 2022-08-26

**Authors:** Kadri Sirkas, Anne Juppo, Mirella Miettinen, Mia Siven

**Affiliations:** a Division of Pharmaceutical Chemistry and Technology, Faculty of Pharmacy, University of Helsinki, P.O. Box 56 (Viikinkaari 5E), FI-00014 Helsinki, Finland; b SIA Novartis Baltics, Pärnu mnt 141, 11314 Tallinn, Estonia; c University of Eastern Finland, Law School, P.O. Box 111, FI-80101 Joensuu, Finland; d Helsinki Institute of Sustainability Science, HELSUS, Yliopistonkatu 3, 00100 Helsinki, Finland

**Keywords:** Paperless package leaflet, Patient information leaflet, Medicine information, Environmental sustainability

## Abstract

**Background:**

Package leaflet provides information about medicinal product to the user. Printed leaflet is familiar and available, however poorly legible, especially when containing multiple languages. It is resourceful to update, has potential to go missing or get damaged, and is environmentally burdensome. The pharmaceutical manufacturers in the Baltic countries have been granted permission to market selected hospital medicinal products without printed package leaflet. The industrial pilot project is expected to promote availability of medicinal products and patient safety via increased access to medicinal information.

**Objective:**

Only few countries in Europe have derogated from Article 58 of Directive 2001/83/EC. Knowledge about the effects of removal of paper package leaflet from the medicinal product is limited, and related publications are scarce. Current interview study is identifying the obstacles during the implementation of the industrial project, investigating the potential environmental impact, and searching for further opportunities for the package leaflet in development of medicinal products.

**Methods:**

Real-time person-to-person semi-structured interviews with relevant stakeholders were conducted, and transcripts were analysed by content analysis to identify themes.

**Results:**

Results demonstrated general support for removing package leaflet from selected hospital products. Main difficulties of the industrial project regarded the need for clear communication and practical disadvantages of project setup. Main benefits included educational aspect of increasing awareness about product information and strengthened collaboration. Majority of participants felt doubtful about the impact of the industrial project on people's awareness of ecological issues and they admittedly lacked sufficient information on the environmental impact of pharmaceutical packaging.

**Conclusion:**

The removal of paper leaflet could be extended to more products based on the positive feedback for the industrial pilot project. However, it is paramount that the format of electronic product information would need to be enhanced first to improve readability.

## 1 Introduction

The aim of a package leaflet is to provide information about the medicinal product to the user. In the European Union (EU), Directive 2001/83/EC on the Community code relating to medicinal products for human use requires that the precisely structured technical document must be available in all official languages of the Member State where it is marketed and in a physical form within the packaging of the medicinal product (Articles 58–59).^1^

Currently, the package leaflet in paper form presents few advantages to the users: it is familiar to users and available due to legislative requirement. The disadvantages of printed leaflet are numerous: it is difficult to read due to small font and complex language (especially for users with visual impairment or other disability), it creates confusion due to multiple languages on one leaflet and proves challenging to find relevant content. Moreover, it is impossible to detect the changes from previous version on printed leaflet. The physical document is time- and energy-consuming to update, and consequently often outdated that impacts patient safety.^2^ According to the United States Food and Drug Administration, mislabelling was accounted for one of the most common reasons for product recalls.^3^ Furthermore, the package leaflet is available only with purchasing the product and can be troublesome to fold and fit back into the package. Thus, it has potential to go missing or get damaged, and it is environmentally burdensome.^2^^,^^4^

### 1.1 Background

Publications with regards to alternatives to printed package leaflet available to date have been focusing on medication user, namely aspects of patient safety, user-friendliness, and receptivity.^2^ Less research has been done to evaluate the environmental aspect of the transfer from paper package leaflets to electronic format. To transit towards sustainable way of living, the member states of the EU have agreed to reduce their environmental impact across all sectors of economy, including in pharmaceutical industry.^5^

One of the initiatives of pharmaceutical manufacturers to minimize their ecological footprint is to optimize the use of material in the finished product. With the support of the European Commission (EC) and in accordance with the Pharmaceutical Strategy,^5^ a permission to derogate from the Directive 2001/83/EC, allowing to market medicinal products without the printed patient information leaflet in packaging, has been granted to Belgium, Luxembourg, Iceland, Sweden, Spain, and Estonia, Latvia, and Lithuania. In spring 2022, the Baltic countries are in active piloting phase.

The industrial project aims to evaluate whether the medicinal products would be used safely without the paper package leaflet and whether the availability of hospital medicinal products could be improved. Overall, it is expected that the removal of paper leaflet increases availability and flexibility of hospital medicinal products and contributes to environmental sustainability with regards to reduction of paper waste. Furthermore, the industrial project supports experimentation, simplification, and digitalisation.^6^^,^^7^ Knowledge about the effects of removal of paper package leaflet from the medicinal product is limited due to lack of practice, and related publications are scarce. This is the first reported study to date investigating such a pilot project. The findings provide valuable insights for further development of delivery of product information to healthcare professionals and patients.

### 1.2 The purpose and aims of the industrial project

As of January 2022, 9 pharmaceutical companies in the Baltic countries entered the industrial project with 13 medicinal products. The decision of the 3 countries Estonia, Latvia, and Lithuania to deviate from current regulation by removing the package leaflet from selected hospital medicinal products was pioneering, strategic and meaningful, and driven by patient safety centric approach. The aim for the industrial project in the Baltic countries was to investigate if the removal of paper package leaflet would increase the availability of medicines on the market, educate healthcare professionals (HCPs) to find the latest approved product information from online sources, and contribute to environmental sustainability in reducing unnecessary paper waste. The medicinal products involved in the project were those that reach the administering HCP only through hospital pharmacy as it holds a special licence for their activity. Importantly, not all medicinal products that are administered by HCPs, were considered eligible for the industrial project. A clear distinction was made for medicinal products that reach the HCP through another route, i.e., via ambulatory private clinics, nursing homes, health, and general practitioners' centres, who order it from a community pharmacy. Albeit never reaching the hands of patients, those medicines were automatically excluded from the project due to logistical reasoning.

The innovative industrial project studied in this paper is in full alignment with the EU's Pharmaceutical and Digital Strategies.^8^^,^^9^ In a hospital setting, the package leaflet in paper format is not always meaningful to HCPs, as instead, they read the Summary of Product Characteristics (SmPC) available on the health authority's website. Furthermore, the industrial project aims to eliminate innate disadvantages of a printed leaflet explained above.

### 1.3 The objectives of the interview study

Few countries in the EU have derogated from the Directive 2001/83/EC. The learnings from these industrial projects could benefit the whole pharmaceutical industry, relevant stakeholders, including patients, and natural environment.

The perception of subjects taking part in the industrial paperless package leaflet project were surveyed in an interview study. The objectives of the study, more specifically, were:

- to identify the obstacles during the implementation of the paperless package leaflet pilot project in the Baltic countries;
- to investigate the potential environmental impact, measured by amount of paper waste by the removal of package leaflets from the packages;
- to search for further opportunities for the paper leaflet in development of medicinal products.

## 2 Methods

For this interview study investigating the industrial project, real time person-to-person semi-structured interviews using open-ended questions, were conducted. Interview study was the preferred research method as it enabled better explanation of the subjects' opinions and experiences with regards to removal of paper package leaflet. The insights collected from the participating experts with the qualitative method provided valuable understandings from different subject matter expert perspectives. The questions were designed to address 3 main topics of the study: industrial project implementation, environmental sustainability, and future opportunities. The questions were the same for every participant and the participants' anonymity was guaranteed. Two pilot interviews with relevant subject matter experts were done prior to data collection to ensure the quality, functionality, and depth of the questions, whereafter a few questions were modified to enhance comprehension. The responses from pilot interviews were not included into the data set.

The participants were recruited to the interview study according to the criteria of being a subject matter expert and involvement in the paperless package leaflet hospital product project. During December 2021 and January 2022, the participants were initially called or emailed to ask for preliminary consent to the study. Thereafter, participants were sent a notice detailing the study and the rights of the participant one week before conducting the interview. They were to read, sign and send the notice back to the researcher before the initiation of the interview, thus providing informed consent to participate. Due to the small size of the industrial project and limited number of involved stakeholders, it was decided to engage the respective subject matter experts to generate in-depth, multi-faceted understanding of different aspects of the project. The data were collected using the virtual platform MS Teams Meeting during February 2022. Interviews were recorded and transcribed verbatim. Obtained transcripts were analysed using content analysis to identify themes.

**Fig. 1:**
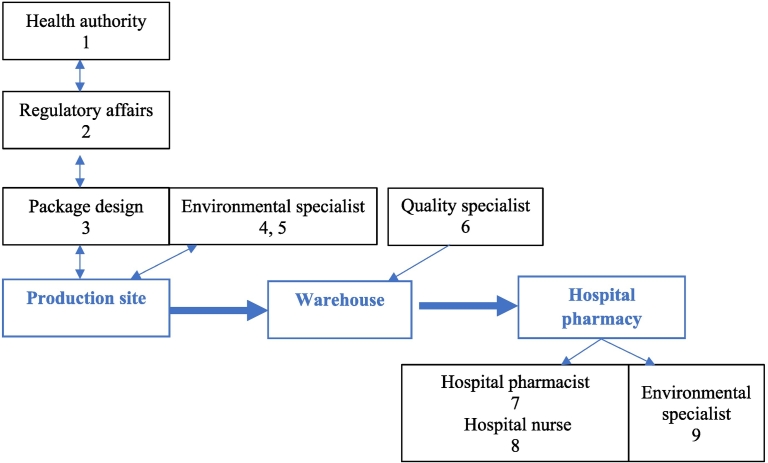
Journey of patient information leaflet with involved stakeholders. Numbers indicate the interviewees (*n* = 9).

Most of the participants in the study were working with the industrial project and were chosen to cover the entire journey of paperless patient leaflet package from regulatory decision until end-user in the hospital, involving national health authority, drug regulatory affairs at pharmaceutical company, drug production site, quality department at pharmaceutical company's warehouse, hospital pharmacy, and hospital nursing department. To investigate the environmental impact of the industrial project, specialists from environmental sustainability departments at pharmaceutical company and at hospital were also interviewed (Fig. 1). Every targeted subject matter expert agreed to participate in the study, resulting in a 100% response rate. The participants were not given the questions before the interview; however, they were informed that the questions will entail the abovementioned 3 research topics. Five interviews were done in Estonian and 4 in English with medium duration of 38 min.

The recordings of the interviews were transcribed, and the transcripts sent back to the interviewees for verification. In this validation phase of the transcript, the participants were able to provide additional explanations and comments to clarify the content, if deemed necessary. Data from the interview transcripts were coded and categorized by themes using software Atlas.ti (ATLAS.ti Scientific Software Development GmbH, Germany).

The data in this study were collected, stored, and handled according to data protection instructions and IT solutions by the University of Helsinki. Data protections notice and informed consent were provided for participants prior to data collection. Data obtained from the interviews were pseudonymous. Informed written consent was obtained, and the participants received verbal and written information of the study. The participants were given information about voluntary participation and the possibility to withdraw it at any time. According to the guidelines of the Finnish National Board on Research Integrity TENK, an ethics review was not required for this study.^10^

## 3 Results and discussion

### 3.1 Industrial project implementation

The key elements of the industrial project in the view of each stakeholder are presented in Table 1.

**Table 1:** Key elements of the industrial project according to function.

Stakeholder	Key elements of the industrial project
Health authority	Coordination of collaboration between 3 countries
	Finding suitable compromises
	Agreeing on how to evaluate results
	Collecting feedback
Regulatory affairs in pharmaceutical company	Collaboration
	Harmonized thinking among regulatory affairs, logistics, wholesalers, quality assurance, production site and health authority
Production site	Meticulously follow instructions from the country regulatory affairs
	Ensure production line readiness
Quality assurance in pharmaceutical company	Proactive participation in cross-divisional team
	Timely review of artwork
	Close checking of packaging components during release
Hospital pharmacy	Sufficient notification of staff
Healthcare professional in hospital	Educate healthcare professionals on where to find the medicinal information online
Environmental specialists	Enable to measure the environmental impact

All 9 participants felt generally supportive for the removing of package leaflet from the product package. The statements in favour mentioned the suitable setting for such an initiative, as the package leaflets of strictly hospital medicinal products rarely reach the hands of the patient and are handled by HCPs who possess necessary medicinal information and are more inclined to looking at the online medicines' registry. It was outlined, that these carefully chosen medicines for the industrial project provide a great starting point to test how the paperless package leaflet medicinal product would work in practice. Therefore, the industrial project presents itself as a useful learning opportunity by implying which products could be added to the potential project extension. Two interviewees were hoping that in the future, legislation would change and packages would not contain printed leaflet as a common practice. In cases where printed leaflet needs to be inserted into the medicinal package, a permission for derogation from the legislative act would be required.

Other supportive statements expressed concerns regarding excess unnecessary paper waste and rationality of the paper leaflet. If the package leaflet is not to be consulted and is discarded from the package directly to trash, it proposes a question to the manufacturers of why add anything to the package that is not needed by the user? It was agreed that formation of waste in healthcare is to some degree inevitable, however efforts, even small ones, should be made to reduce waste wherever possible, as in time they add up. The industrial project falls well under the various environmental sustainability plans within organisations, therefore finding support among interviewees as an actionable initiative. One of the interviewees concluded:

*“It is not that we are getting rid of it, and we cannot have a printed paper leaflet again. It is more about printing when you need it as opposed to printing it all the time”.*

Additionally, the initiative was welcomed in terms of digitalization and future trends. Pharmaceutical companies, as well as the EC and local health authorities have established digital strategies, and the industrial project fits well into these agendas.^8^^,^11., 12., 13 Tendency to go digital in manufacturing, product release and approval processes, and document management systems with validated interlinked systems and electronic signatures is increasingly acceptable by competent authorities.^13^ Interviewees mentioned that the people, especially HCPs, are generally well-accustomed to the internet and digital platforms. The industrial project was perceived as the first step forward towards introducing users to available online leaflet information.

The industrial project was also encouraged from the perspective of artwork creation, as a significant part of the artwork production activities is to change the leaflets according to the constant updates. The implementation of these amendments is often highly regulated, strictly time-critical, resource intensive and adds complexity to the process. Any type of variation is an interruption to the production process, resulting in approximately 9 months to get updated product information through the whole supply chain to patients. Therefore, removing the package leaflet in paper format where not needed, allows smoother production flow, wiser use of resources and shorter lead time onto market.

The paperless package leaflet project especially benefits the products for joint markets, such as Estonia, Latvia, and Lithuania. Common Baltic presentation with 3 languages appearing on packaging material results in voluminous package leaflets, which are difficult to handle and read.^14^ Furthermore, the alignment of the requirements and approvals of the 3 health authorities adds complexity to the artwork creation and production process. With regards to the size of package leaflets, the interviewees working at the hospital brought out a daily issue - leaflets, once taken out from the pack, are difficult to put back in and hinder the repositioning of the medicinal product back to the pack. Thus, the administering staff are prone to removing the leaflet from the pack already before going to the ward.

Patient safety was mentioned as an important supportive aspect of the industrial project, as electronic leaflet can prevent the use of outdated information. Package leaflet is an official document of marketing authorisation, that is constantly changing in time and the marketing authorisation holder should implement the amendments into leaflet relatively fast. In search of medicinal information, the trustworthy source with validated documents is to be found on the webpages of the national state agency of medicines.15., 16., 17. Participants highlighted that source of latest approved product information should be consulted as a habit by HCPs, as medicines information cannot and should not be memorized.

Nonetheless, some opposing concerns were shared about the industrial project. Although the medicines used in hospital setting are generally well-known and habitual to HCPs, there are some instances where the printed package leaflet could be useful, such as for new employees or in case of a novel medicine. It was emphasized that even though the medical use of the medicine itself is known to the HCP, information about its storage, handling and preparation is not. As this important piece of knowledge is not printed on the pack, it might cause issues with proper management of the medicine. Therefore, with the removal of the package leaflet in the pack, essential information about the medicine should be readily available together with the medicinal product, even if it is retrievable online.

Furthermore, it was mentioned that the layout of package leaflet is not user-friendly and the replacement of leaflet in paper format with electronic static pdf-file does not enhance its readability. Another concerning factor was the lack of or restricted access to internet (mobile) platform or the HCPs inability to use it. Finally, with regards to the product information available online for centrally registered products in the EU, the files are lengthy, consisting of SmPC, labelling, additional obligations for the marketing authorisation holder, and, only in the end, the leaflet. The big size of the file creates confusion and makes it difficult to navigate.^2^^,^^4^^,^^18^^,^^19^ An easy way to modify the form of the electronic product information is to include a navigation pane to the file so that the specific product information could be easily found.

**Fig. 2:**
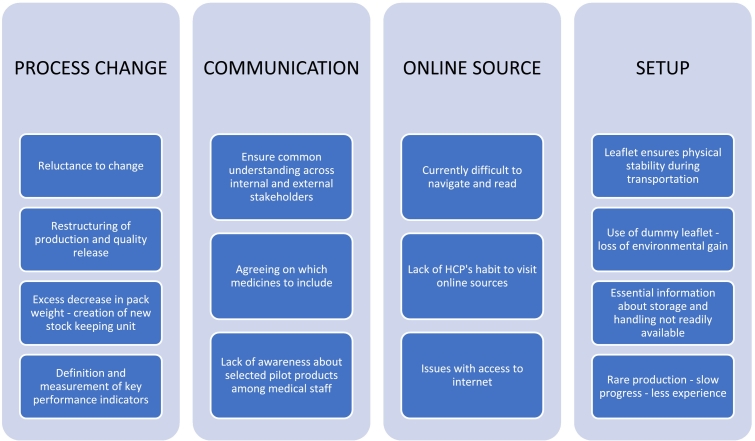
Difficulties of the paperless package leaflet industrial project.

**Fig. 3:**
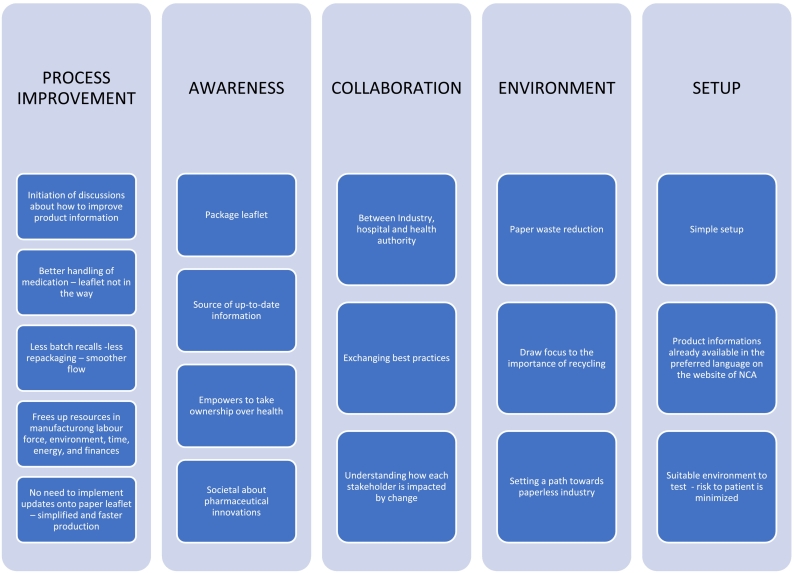
Benefits of the paperless package leaflet industrial project.

Fig. 2, Fig. 3 present the potential problems and benefits that could arise during the industrial project implementation. The interview study discovered that the main difficulties included: issues with process change, ensuring clear communication, difficulties using the online source for product information, and practical disadvantages of project setup.

In this interview study several benefits of the industrial project were identified, such as process improvement, educational aspect of increasing awareness, strengthened collaboration, environmental benefit, and practical advantages of the project setup.

With regards to acceptance of electronic sources of information, previous research has been done among medicine users. According to the study conducted among 406 patients in 2014 by Hammar et al., 41% of the respondents felt positive towards reading the patient information leaflet electronically, whereas 32% felt hesitant and 26% neutral.^2^ The recent results from the first round of paperless leaflet hospital product project from Belgium and Luxembourg demonstrate that the digital format of the leaflet is preferred by the HCPs with 98% of the pharmacists agreeing to remove the paper leaflet from the packaging of medicinal products restricted to hospital use.^20^^,^^21^

### 3.2 Environmental sustainability

As the industrial project included only 13 products by 9 companies for 3 small markets, the environmental impact of the project was considered non-substantial. On one-package level, if the leaflet from each of the selected hospital medicinal products in the participating countries is removed, it adds up to estimated total of 279 pages of leaflet. The number of leaflets left unprinted in the project would be multiplied by the quantity of medicines produced during the 2-year industrial project period.15., 16., 17. However, its value being principal and future-looking, the environmental aspect was generally regarded important in changing the thinking about how to operate in burdening the environment as little as possible. If people are educated and encouraged to use trustworthy electronic sources for information instead of printing them out by default, it creates an impact on mindset that will cross over to other areas of life. The results showed a general lack of information among participants on the environmental impact of pharmaceutical packaging, indicating the need for accessible and reliable data.

In case of a successful industrial project and scaling it up to more products or markets, however, the impact would be significant, participants concluded. As the size of a leaflet varies greatly depending on the medicine and country's regulatory requirements, sometimes reaching to size measured in metres, the amount of paper saved by removing the leaflet could be remarkable in some instances. In addition, when reducing the package size, more medicines could be transported per pallet and per vehicle, resulting in an even bigger environmental gain. Furthermore, the material of package leaflets might cause difficulties in recycling as they can be glossy, thick, booklet-type with metal clips, etc. In that light the industrial project might open discussions on type of material for package inserts.

Removing package leaflet from the package – where it is not needed – was generally considered the right thing to do regarding environmental sustainability. As of one participant argued:

*“If we just go and talk to the hospitals, go, and ask them what they do with their leaflet? Do they take each one of them out, read them, write down all the stuff or do they just take it and throw it away? That answers the whole question”.*

Broadly, there is the paper itself that will not be produced plus all the related activities to have the package leaflet produced (ink, printing houses, special thin type of paper, etc). Manufacturers feel uncomfortable producing something that is harmful to the environment, especially if the HCPs do not need it, making the leaflet in this setting “a meaningless waste”, participants stated. There is a clear connection established between human and planetary health.^22^^,^^23^ Participants stated it to be an equally beneficial situation if the users have access to most up-to-date product information while avoiding unnecessary paper leaflets being produced and distributed to hospitals who then need to manage the waste.

Environmental considerations are important in the industrial project, but they must go hand in hand with regulatory requirements that ensure patient safety, i.e., all essential information about the storage and handling must be present on the package. It was commonly agreed that no compromises can be made over the welfare of patients, but they must serve each other. According to one participant:

*“This [industrial project] is not about hiding information, but about improving availability of information.”*

With regards to the worsening climate situation worldwide, it was considered ethically fair to implement measures to avoid depletion of natural resources, as population health also depends on clean environment. Participants concluded that in this project *“you run a marathon”* and *“every step counts”.*

There has been little research on the opinions and environmental aspect of the transition from paper to electronic package leaflets. A recent publication revealed the attitudes towards pharmaceuticals-related environmental issues among Finnish population.^24^ Most of the respondents were concerned about the environmental and health impact of pharmaceutical residues and emphasized topics such as the importance of environmentally sustainable actions by pharmaceutical companies and the recyclability of the pharmaceutical packaging materials. Another study demonstrated a high public support to establish environmentally friendly pharmaceutical policies, and the support was in proportional correlation with population's environmental attitude.^25^ This concludes that the public is open to and expects to discuss environmental sustainability in healthcare.

It was also highlighted that processes necessary for the production and distribution of the medicines are getting digitalized, signatures move from wet to electronic, and the accompanying documentation is stored virtually. It is assumed that the Covid-19 pandemic since 2020 has accelerated the transition from paper documentation to electronic file management systems.^22^^,^^26^

Participants were asked to discuss where else one could optimize paper consumption in medicinal packages. They listed reducing outer packages (by removing package leaflet, smaller immediate packaging, or less obligatory text on outer carton), recycling and reusing packaging material and shortening the package leaflet. It was stated that the size of medicines in comparison to their excess packaging seem disproportionate and a few participants were wondering why some of the blister holes are kept empty. For the medicinal products that are inside a bottle, jar, or some other container, it should be carefully considered if an additional outer carton package is indeed necessary. One of the interviewees was arguing whether the requirements regarding Falsified Medicines Directive were the reason to add a carton outer package to medicines in bottles.^1^^,^^27^^,^^28^ The idea to print the package leaflet as per individual need was brought out by several participants, however it was admitted that the system should be given thorough thought as to who, when and how to provide the printout. From the perspective of hospital use for frequently used medicines, multipacks, i.e., packages with large quantities of medicinal product inside should be encouraged during procurement.

Hospital pharmacist and/or nurse in the hospital ward receiving the medicinal product without leaflet will be most influenced by the change, as.

*“…we will have one thing less to discard”.*

Majority of participants were doubtful about the impact of the industrial project on people's awareness regarding environmental issues. It was assumed that the potential effect on environmental awareness is person-specific, impacting those who are already ecologically conscious. On the other hand, it was stated that the missing leaflet might cause users to think that it is an initiative of pharmaceutical company to cut manufacturing cost or simply a production error. Some of the interviewees believed the missing leaflet will not be even noticed. Nonetheless, the concomitant positive ecological outcome of the industrial project was regarded as an additional – “certainly not insignificant” – result.

### 3.3 Future opportunities

Third theme of the interview surveyed the future directions for development of patient leaflet. Participants felt that they did not have sufficient information and that they did not trust the information available on environmental sustainability. It was pointed out that the calculations are unfortunately dubious, rounded, and difficult to comprehend by majority of people. One participant observes:

*“You could have the same thing done twice with two different routes and you come up with two different answers”.*

Numbers describing the environmental effect should be calculated based on trustworthy transparent sources, avoid under- or overstating, and described in meaningful terms, e.g., what is the number of football fields if spreading out the printed leaflets per year. To make the message effective, the environmental savings gained should be calculated and translated into relatable language.^29^

**Fig. 4:**
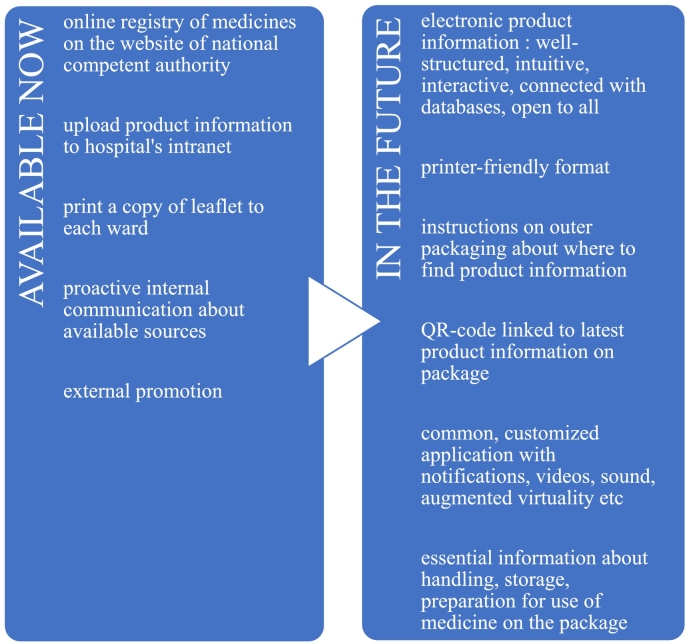
Compensation for the missing paper leaflet with means available now and in the future.

Fig. 4 compiles the participants' suggestions on how best to compensate for the missing paper leaflet with means made available now and, also, in the future.

In case the industrial project receives positive feedback, it could be extended to more products. However, participants think it is paramount that the format of electronic product information should be enhanced first. According to the knowledge of the interviewees, efforts are being made across the world to create platforms for user-friendly, technologically advanced, and reliable consumption of medicinal information, such as Electronic Product Information (ePI), Electronic Leaflet (eLeaflet) and Electronic Patient Information Leaflet (ePIL).^6^^,^^14^^,^^20^^,^^21^ These applications enable health authorities, manufacturers, HCPs, and patients/users to connect.

The medicinal products that could be included in the next phases of the paperless package leaflet project are presented in Table 2. The answers show the potential to extend the project gradually onto many types of medicines based on the premise that risks are well mitigated.

**Table 2:** Medicinal products that could be included in the next phases of the industrial project.

Order of inclusion into project	Medicinal product type	Comment
1.	Left out from the first round	Via community pharmacies directly to administering healthcare professional
2.	With accompanying patient brochure	As part of additional risk minimisation measure
3.	Widely used in hospitals	E.g., anticoagulants, analgesics, etc
4.	For chronic use	E.g., medicinal products to lower blood pressure, glycose, and cholesterol
5.	With wide range of indications	To avoid confusion among patients about the application
6.	Most prescription medicinal products	Except more dangerous medicines, with serious adverse drug reactions, one-time use only, chirurgical use, etc
7.	Over-the-counter medicinal products	Begin with most frequently used Information available in smart devices On-demand printouts

Importantly, when discussing which medicines to include into the potential second round of the industrial project, it was encouraged to consult patient advocacy groups, as they are most familiar with their situation and needs.^2^^,^^30^^,^^31^

The debate on the use of technology to support innovation in healthcare is widespread. The EU has declared the following decade as the Digital Decade in pursuing a human-centric and sustainable vision for digital society.^9^ Hämeen-Anttila et al. (2018) revealed in their study that internet is already a frequent source of medicines information.^32^ To better meet the needs of patients and HCPs, European Federation of Pharmaceutical Industries and Associations (EFPIA) has stated a position on shortcomings of SmPC and package leaflet with proposals to resolve them. In support of providing comprehensive, accurate and up-to-date information on medicinal products to HCPs and patients, EFPIA states it must be easily accessible and adjustable to the need of the individual. Alternative dissemination methods, such as internet or mobile devices or direct printouts at dispensing level, should be explored, as it would facilitate updates in product information. It is important to have the information in a user-friendly structure and ensure users have access to trusted source of authorised product information. Furthermore, electronically available systems could better meet the need of disabled people or people with specific requirements of information representation.^33^ To improve user-friendliness of medicinal information, a chat bot type of conversational agent for querying package leaflet has been created. This system simplifies health information retrieval and improves health literacy in Italian by codifying leaflets and making them query-able in natural language.^34^

Package leaflet in medicinal products is subject to improvement. Its physical presence could be made optional, and its content could be improved to increase readability, user-friendliness, and comprehensibility.

### 3.4 Limitations

Despite the research design aiming to study key stakeholders in the industrial project, the study results cannot be generalized as the number of participants is low (9 interviewees). However, this was due to the small size of the industrial pilot project (9 companies with 13 medicinal products) and limited number of stakeholders working in the project in the Baltics. As few of the respondents were directly involved in the planning of the industrial project, it is fair to assume there could be some bias in the responses. Still, they were considered as the experts in this research area and therefore important subjects for interviews. Additionally, during the interviews the first medicinal products without printed package leaflet had not yet arrived to market, resulting in responses being theoretical in nature. Furthermore, the study was conducted in the Baltic countries, which are technologically advanced and more prone to utilizing digital tools, thus the results cannot be applied to every setting.

## 4 Conclusion

Package leaflet provides information about the medicinal product. Printed leaflet is familiar to users and readily available when purchasing the product, however, demonstrates disadvantages such as poor usability and being environmentally burdensome. The removal of paper leaflet from carefully selected hospital products is pioneering to explore how the availability of medicinal products could be improved and how best to communicate product information to users.

Generally, the idea of removing package leaflet in paper form was well accepted, however it was recommended that the format of electronic product information would be enhanced first. This could be done by including a navigation panel to the file to improve searchability, for example, and by maximising digital opportunities. The results demonstrated potential to extend the industrial project to more medicinal products. Although the reduction of paper waste was welcomed, the impact of the industrial project on awareness about environmental issues was deemed doubtful. The respondents' responses revealed a general lack of information on the environmental impact of pharmaceutical packaging. Broader studies on the impact of paperless package leaflet on various stakeholders and in different settings are needed.

## Role of the funding source

This research did not receive any specific grant from any funding agencies in the public, commercial, or not-for-profit sectors.

## Declaration of Competing Interest

None.

## Appendix A Supplementary data

Supplementary material Image 1

## References

[1] European Commission Directive 2001/83/EC of the European Parliament and of the Council of 6 November 2001 on the Community code relating to medicinal products for human use. https://ec.ehttp://data.europa.eu/eli/dir/2001/83/oj.

[2] Hammar T., Nilsson A.L., Hovstadius B. (2016). Patients’ views on electronic patient information leaflets. Pharm Pract.

[3] Hall K., Stewart T., Chang J., Freeman M.K. (2016). Characteristics of FDA drug recalls: a 30-month analysis. Am J Health-Syst Pharm.

[4] Pander Maat H., Lentz L. (2010). Improving the usability of patient information leaflets. Patient Educ Couns.

[5] European Commission The European Green Deal. https://ec.europa.eu/info/strategy/priorities-2019-2024/european-green-deal_en.

[6] Republic of Estonia Agency of Medicines Estonian, Latvian and Lithuanian agencies announce the ePIL project for hospital use medicines. https://www.ravimiamet.ee/en/news/estonian-latvian-and-lithuanian-agencies-announce-epil-project-hospital-use-medicines.

[7] The Association of Pharmaceutical Manufacturers in Estonia (2022). Muutuse Võimalus - Paber-Infoleheta Haiglaravimid.

[8] European Commission A Pharmaceutical Strategy for Europe. https://ec.europa.eu/commission/presscorner/detail/en/ip_20_2173.

[9] European Commission Europe's Digital Decade. https://digital-strategy.ec.europa.eu/en/policies/europes-digital-decade.

[10] Finnish Advisory Board on Research Integrity (2019). Finnish National Board on Research Integrity TENK Guidelines 2019: The Ethical Principles of Research with Human Participants and Ethical Review in the Human Sciences in Finland. https://tenk.fi/sites/default/files/2021-01/Ethical_review_in_human_sciences_2020.pdf.

[11] Novartis Data and Digital. https://www.novartis.com/about/strategy/data-and-digital.

[12] European Federation of Pharmaceutical Industries and Associations Digital health: The power behind. https://www.efpia.eu/about-medicines/use-of-medicines/healthcare-systems/digital-health/.

[13] European Medicines Agency (2020). https://www.ema.europa.eu/en/documents/other/european-medicines-agencies-network-strategy-2025-protecting-public-health-time-rapid-change_en.pdf.

[14] European Medicines Agency EMA action plan related to the European Commission's recommendations on product information. https://www.ema.europa.eu/en/documents/other/european-medicines-agency-action-plan-related-european-commissions-recommendations-product_en.pdf.

[15] Estonian Agency of Medicines Registry of Medicines. https://ravimiregister.ee/.

[16] Latvian Agency of Medicines Registry of Medicines. https://dati.zva.gov.lv/zalu-registrs/en.

[17] Lithuanian Agency of Medicines Registry of Medicines. https://vapris.vvkt.lt/vvkt-web/public/medications.

[18] Young A., Tordoff J., Smith A. (2017). ‘What do patients want?’ Tailoring medicines information to meet patients’ needs. Res Soc Adm Pharm.

[19] Mühlbauer V., Prinz R., Mühlhauser I., Wegwarth O. (2018). Alternative package leaflets improve people’s understanding of drug side effects—a randomized controlled exploratory survey. PLoS One.

[20] Association Generale de l'’Industrie du Medicament, European Agency of Medicines (2018).

[21] eLeaflet An e-PIL pilot project in Belgium & Luxembourg. More Eu countries experimenting with eLeaflet solutions. https://blog.eleaflet.eu/en/an-e-pil-pilot-project-in-belgium-and-luxembourg.

[22] de León E.A., Shriwise A., Gö Tomson (2021). Beyond building back better: imagining a future for human and planetary health. Lancet Planet Health.

[23] Wyns A., Beagley J. (2021). COP26 and beyond: long-term climate strategies are key to safeguard health and equity. Lancet Planet Health.

[24] Alajärvi L., Timonen J., Lavikainen P., Martikainen J. (2021). Attitudes and considerations towards pharmaceuticals-related environmental issues among finnish population. Sustainability (Switzerland).

[25] Alajärvi L., Lehtimäki A.V., Timonen J., Martikainen J. (2022). Willingness to pay for implementation of an environmentally friendly pharmaceutical policy in Finland—a discrete choice experiment study. Int J Environ Res Public Health.

[26] Quintana A., Venkatraman R., Coleman S.B., Martins D., Mayhew S.H. (2021). COP26: an opportunity to shape climate-resilient health systems and research. Lancet Planet Health.

[27] Oujo W. The challenge of packaging compliance to the Falsified Medicines Directive. http://packaging.stdy.org/the-challenge-of-packaging-compliance-to-the-falsified-medicines-directive/.

[28] European Commission (2011). Directive 2011/62/EU of the European Parliament and of the council of 8 June 2011 amending directive 2001/83/EC on the community code relating to medicinal products for human use, as regards the prevention of the entry into the legal supply chain of falsified medicinal products. Off J Eur Union.

[29] de Feo G., Ferrara C., Iannone V., Parente P. (2019). Improving the efficacy of municipal solid waste collection with a communicative approach based on easily understandable indicators. Sci Total Environ.

[30] Patient-Focused Medicine Making systematic patient engagement a reality across medicine and device lifecycles, digital health, data and health systems for better outcomes. https://patientfocusedmedicine.org/.

[31] Algabbani A.M., Alzahrani K.A., Sayed S.K. (2022). The impact of using pictorial aids in caregivers’ understanding of patient information leaflets of pediatric pain medications: a quasi-experimental study. Saudi Pharm J.

[32] Hämeen-Anttila K., Pietilä K., Pylkkänen L., Pohjanoksa-Mäntylä M. (2018). Internet as a source of medicines information (MI) among frequent internet users. Res Soc Adm Pharm.

[33] European Federation of Pharmaceutical Industries and Associations (2014). Position on Shortcomings of the Summary of Product Characteristics and the Package Leaflet and Proposals to Resolve Them. https://www.efpia.eu/media/288342/position-on-shortcomings-of-the-summary-of-product-characteristics-and-the-package-leaflet-and-proposals-to-resolve-them.pdf.

[34] Minutolo A., Damiano E., de Pietro G., Fujita H., Esposito M. (2022). A conversational agent for querying Italian patient information leaflets and improving health literacy. Comput Biol Med.

