# Supplementary material for: Could paper package leaflet be left out from hospital products?
Source: Explor Res Clin Soc Pharm. 2022 Aug 26;7:100176. doi: 10.1016/j.rcsop.2022.100176 (PMC9465427; doi:10.1016/j.rcsop.2022.100176)
Supplement: Supplementary file 1 — Supplementary material [file mmc1.docx]

1. Pilot project implementation

**What are the obstacles of the paperless package leaflet hospital product pilot project implementation?**

- 1. What do you think about the idea of removal of package leaflet from the product package? Do you see the need for this type of pilot project?
  2. What kind of problems could arise during the pilot project implementation?
  3. What are the potential benefits?
  4. Within your function, what is key to ensuring a smooth paperless package leaflet project implementation?

1. Environmental sustainability

**What is the environmental impact of the pilot, measured by the amount of paper waste by the removal of package leaflets from the packages?**

- 1. Transitioning towards digital solutions in healthcare, the focus is on patient safety. Please explain, how important in your opinion is the environmental aspect of the pilot project?
  2. In your opinion, is the removal of the package leaflet from the package the right thing to do regarding environmental sustainability? Explain, why/why not.
  3. Where else could we look for to optimize paper consumption in medicinal packages?

1. Future opportunities

**What are further opportunities for the paperless package leaflet?**

- 1. What is the influence of the paperless package leaflet project on awareness about environmental issues for the users?
  2. How best to compensate for the missing paper package leaflet for the user with means available in the moment?
  3. How best to compensate for the missing paper package leaflet for the user with means made available in the future?
  4. In case of a successful paperless package leaflet project, would you imagine broadening it to more medicinal products? What could the next extensions be?
